# Understanding the Physiological Role of Electroneutral Na^+^-Coupled HCO_3_^−^ Cotransporter and Its Therapeutic Implications

**DOI:** 10.3390/ph15091082

**Published:** 2022-08-30

**Authors:** Jingjing Wang, Aqeela Zahra, YunFu Wang, Jianping Wu

**Affiliations:** 1 School of Chemistry, Chemical Engineering and Life Sciences, Wuhan University of Technology, Wuhan 430070, China; 2 Hubei Key Laboratory of Embryonic Stem Cell Research, Hubei University of Medicine, Shiyan 442000, China

**Keywords:** Na^+^/HCO_3_^−^ cotransporter, central nervous system, pH, cardiovascular system, digestive system, kidneys, cancer

## Abstract

Acid–base homeostasis is critical for proper physiological function and pathology. The SLC4 family of HCO_3_^−^ transmembrane cotransporters is one of the HCO_3_^−^ transmembrane transport carriers responsible for cellular pH regulation and the uptake or secretion of HCO_3_^−^ in epithelial cells. NBCn1 (SLC4A7), an electroneutral Na^+^/HCO_3_^−^ cotransporter, is extensively expressed in several tissues and functions as a cotransporter for net acid extrusion after cellular acidification. However, the expression and activity level of NBCn1 remain elusive. In addition, NBCn1 has been involved in numerous other cellular processes such as cell volume, cell death/survival balance, transepithelial transport, as well as regulation of cell viability. This review aims to give an inclusive overview of the most recent advances in the research of NBCn1, emphasizing the basic features, regulation, and tissue-specific physiology as well as the development and application of potent inhibitors of NBCn1 transporter in cancer therapy. Research and development of targeted therapies should be carried out for NBCn1 and its associated pathways.

## 1. Introduction

The maintenance of intracellular pH (pHi) and extracellular pH (pHo) is critical for biological function. The fluctuations in pH affect the enzymatic activity, as well as the function of the cell membrane and the signaling molecules, which inevitably contribute to modifications in cellular activity [1]. The Na^+^/H^+^ exchangers and the Na^+^-dependent/independent HCO_3_^−^ transporters are the primary transporters that are responsible for mediating net acid extrusion in the majority of mammalian cells. HCO_3_^−^ is one of the most essential acid–base buffer ions and is involved in regulating the pH of two gene families in vertebrates, invertebrates, and humans (SLC4 and SLC26) [2].

SLC4 consists of 10 genes, nine of which encode HCO_3_^−^ cotransporters that are either Na^+^-independent (AEs) or Na^+^-dependent (NBCs) [3,4]. Until now, SLC4 family members are the only molecular entities functionally shown as Na^+^-dependent HCO_3_^−^ transporters. The Na^+^-dependent HCO_3_^−^ transporters are further divided into electrogenic cotransporters NBCe1 (SLC4A4) and NBCe2 (SLC4A5), electroneutral cotransporters NBCn1(SLC4A7) and NBCn2(SLC4A10), electrically neutral Na^+^-driven Cl^−^/HCO_3_^−^ exchanger (NDCBE) [5,6]. Despite their obvious uniformity of more than 30% amino acid sequence identities between these proteins, NBCs vary in three major aspects, for example, ion selectivity, transportation pattern, and cellular localization.

**Figure 1:**
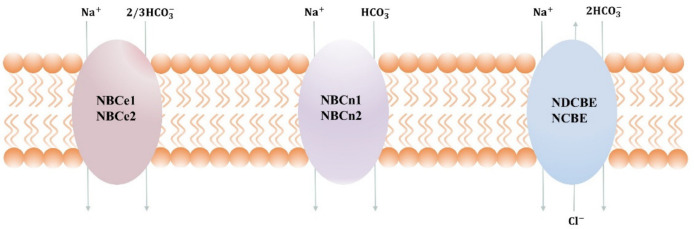
Transport stoichiometry of NBCs at intracellular (I) and extracellular (o) ion concentrations and membrane potentials. Note that NBCn2 and NCBE are the two alternative names suggested for SLC4A10, depending on whether the transporter mediates net Cl^−^ transport or only Cl^−^ self-exchange.

The NBCs family is essential in the regulation of pH and the transdermal release and uptake of Na^+^, HCO_3_^−^, and Cl^−^ in different tissues [7,8]. The mode of transport, along with membrane potential and ion gradient, is a key factor in determining the direction of transport. At resting membrane potential and normal ion gradient, HCO_3_^−^ and Na^+^ are transported 1:1 or 2:1 into the cell and transported 3:1 out of the cell. In some pathophysiological conditions, such as ischemia, cancer, etc., the transmembrane ion gradient and membrane potential change significantly, which may affect the flow of transport as well as the activity of acid–base cotransporters. Within the cytosol and interstitial space near the transport site, the transmembrane transfer of HCO_3_^−^ occurs in the chemical reaction CO_2_+H_2_O⇔HCO_3_^−^+H^+^, which is catalyzed by carbonic anhydrase [9]. When equilibrium is reached, the transport of HCO_3_^−^ is equivalent to the transport of H^+^ in the opposite direction, as shown in Figure 1. NBCn1 transports Na^+^ and HCO_3_^−^ with a stoichiometry of 1:1, whereas NBCe1 and NBCe2 can transport with a stoichiometry of 1:2 or 1:3, depending on the cell type and phosphorylation status [10]. NCDBE facilitates electroneutral Na^+^-dependent Cl^−^/HCO_3_^−^ exchange. It remains controversial whether the Na^+^-dependent HCO_3_^−^ transport mediated by NCBE/NBCn2 is coupled to net Cl^−^ transport or whether it is solely related to Cl^−^ self-exchange.

Since each member of the NBCs family is expressed in a variety of cell types, the disruption of even a single gene may often result in severe and intricately linked clinical manifestations, as shown in Table 1. However, there are not many clinical reports or tissue samples available from people who have been afflicted with these rare diseases, which makes it difficult to comprehend their underlying causes and develop the appropriate therapies.

Among all the NBCs family members, the role of electroneutral Na^+^/HCO_3_^−^ transporter (NBCn1) in acid–base homeostasis is indispensable and studied as a master regulator of cellular acid extrusion [17]. Understanding the basic structure, regulation, and physiology of NBCn1 is essential for assessing its significance as a therapy target and developing a drug treatment that is optimally harmless and effective. The present review aims to provide a comprehensive summary of the latest advancement in the study of NBCn1, focusing on the fundamental characteristics, regulation, and tissue-specific physiology as well as the development and application of potent inhibitors of NBCn1 transporter in cancer treatment. Since NBCn1 cause a net outflow of H^+^ and an inflow of Na^+^, they are of apparent importance in cancer research and therapy.

## 2. NBCn1

**Figure 2:**
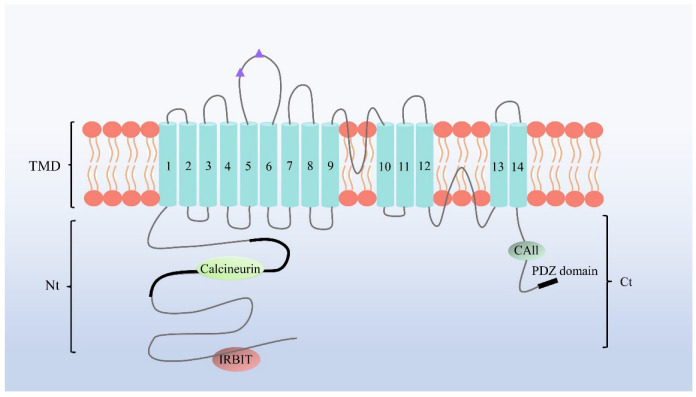
NBCn1 topology. The figure illustrates the predicted NBCn1 membrane topology and localization of the primary binding site, where IP3Rs binding protein released with IP3 (IRBIT) and Carbonic Anhydrase II (CAII) can enhance the activity of NBCn1.

The NBCn1, originally called NBC3, was first extracted from human skeletal muscle cells [18] and mapped to the 3p22 human gene locus [19]. However, the structure of NBCn1 has not been explicitly established since NBCn1 has 55–67% homology with the NBCe1 and 33–43% homology with the AEs [20]. The current understanding of the topology of the NBCn1 transporter protein TMD is mainly from a series of studies performed on NBCe1 and AEs. It is projected to constitute a long N-terminal (Nt) region, 10–14 transmembrane domains, and a small C-terminal (Ct) region in the cytoplasm [21,22]. NBCn1 can bind the carbonic anhydrase II(CAII), and its binding is essential for transport activity; mutation at the binding site reduces HCO_3_^−^ transport activity by 40%. It has been proposed that phosphorylation of the Ct structural domain of NBCn1 can regulate the Na^+^/HCO_3_^−^ coupling ratio by displacing CAII from its binding site [23]. This finding suggests that HCO_3_^−^ transporter protein/CAII interactions can be regulated in a phosphorylation state-dependent manner. In addition, the Ct structural domain of NBCn1 may play a similarly important role, with deletion of Ct reduced transport by nearly 90% [24], suggesting that the Ct is necessary for the transport of NBCn1. The topology of NBCn1 is shown in Figure 2. NBCn1 is homogeneous to other NBCs; when it is dysfunctional, other members of NBCs may compensate for its function [25]. However, due to each transporter protein acting at a different site and having different regulatory mechanisms, it is hard to predict which one will take its role.

**Figure 3:**
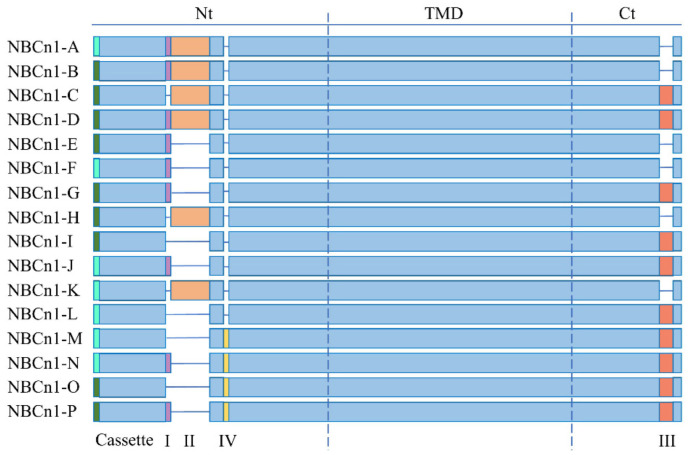
NBCn1 protein variants. The protein sequences contain two different Nt and can be organized into different cassettes by combining variable parts.

Later, researchers cloned three NBCn1 variants from the aorta of rodents (B, C, and D) that are similar to NBC3 and demonstrated that the B variant encodes an electroneutral Na^+^/HCO_3_^−^ cotransporter, named NBCn1-B [21]. In subsequent studies, NBCn1 was found in many tissues in the form of a variety of variants; in total, 16 functional NBCn1 splice variants have been identified, ranging from NBCn1-A to NBCn1-P [7], as shown in Figure 3.

In the NBCn1 variant protein product, the Nt started with MERF or MEAD. Initially, the sequence that began with MERF was reported for human variants, while that began with MEAD was all from rats [26]. At present, it is not clear whether MERF/MEAD represent species differences. Furthermore, these variations are simplified by combining alternative splicing variable sections into the cassette, three of which are found in the Nt cytoplasmic domain (cassette I, II, and IV), and one of which is located in the Ct cytoplasmic domain (cassette III). Cassette I is a sequence of 14 amino acids with unclear functional significance [22]. Splice variations with and without cassette I appear to be co-expressed in the majority of tissues [27]. It was later confirmed that the renal cortex, submandibular glands, parotid glands, and liver appear to have a preference for cassette I inclusion; on on contrary, the lung appears to have a preference for cassette I exclusion [8,26]. There is a 123–124 amino acid sequence in cassette II. Whether NBCn1 with or without cassette II is expressed in Xenopus oocytes, it does not seem to change the transport properties of NBCn1 [28]. There are many potential calcineurin binding sites in cassette II, suggesting it may play a regulatory role in more intricate expression systems [29]. The cassette III is a sequence that is 36 amino acids [22]. NBCn1 splicing appears to favor the inclusion of cassette III in adult mouse organ/tissue preparations. However, there appears to be a preference for the inclusion of cassette III in the renal cortex [27]. The cassette IV appears to have a role in the retina since it can decrease protein-membrane abundance and increase NBCn1 activity [26]. Moreover, interactions between multiple cassettes regulate surface expression; therefore, it is challenging to attribute a particular effect to that of any individual cassette.

## 3. Function of NBCn1

Since NBCn1 expression is found in different types of cells in almost every organ of the body [30], it may have a role in regulating a varied range of biological activities. It is hypothesized that NBCn1 protects cells against intracellular acidification due to its function as a mediator of net inward HCO_3_^−^ transport. In addition, numerous cellular proteins, including enzymes and ion channels, are sensitive to fluctuations in pH, although the effects of intracellular acidification on multifaceted cellular and systemic activities are still largely unknown.

### 3.1. Role of NBCn1 in the Central Nervous System

In central nervous system (CNS), NBCn1 has been found to be expressed in a wide variety of cell types, with particularly strong expression in the hippocampal region, cerebral cortex and cerebellar lobe, and brainstem cells, as well as choroid plexus epithelial cells [30,31,32,33,34]. NBCn1 plays an important role in brain development [35]. In addition, NBCn1 has a critical function in the regulation of physiological pH, primarily in neuronal excitability precisely at synapses [36].

In astrocytes, an action potential increases electrogenic Na^+^/HCO_3_^−^ cotransport [36] and reduces localized pHo. In presynaptic neurons, the low pHo restricts Na^+^-driven Cl^−^/HCO_3_^−^ exchange, which in turn causes the neuronal pHi to become more acidic. Consequently, acidification leads to feedback attenuation of the action potential. Many NBCs transporters at multiple synapses must be activated simultaneously in order to achieve this feedback inhibition [29]. Synaptic NBCn1 localization suggests a role for Na^+^/HCO_3_^−^ transport in neuronal modulation. Any alteration to the pHo that occurs at synapses will most likely have an effect on the activity of NBCn1, which in turn may cause a change in either the pre or postsynaptic pHi. Due to the sensitivity of numerous neurotransmitter receptors and ion channels to pH, including N-methyl-D-aspartate receptors (NMDA) [37], ɤ-aminobutyric acid (GABA) A receptors [38], and voltage-gated Ca^2+^ channels [39], an NBCn1-mediated pHi and/or pHo change would significantly affect synaptic activities.

Numerous studies have demonstrated that astrocytes have essential functions in regulating the extracellular concentrations and neurotransmitters of ions, as well as protecting neurons from excitotoxic and oxidative insults. In the hippocampus, NMDARs that are sensitive to Mg^2+^ can be blocked by Mg^2+^ in a voltage-sensitive and non-competitive manner. The Mg^2+^ acts as a cofactor for ATP and enzymatic reactions, as well as a regulator of ion channels and receptors, decrease in Mg^2+^ concentration reflects the severity of neuronal injury [40,41]. However, NMDARs are involved in cytotoxicity under Mg^2+^-free conditions, mediating intracellular calcium ions load and triggering apoptosis. It has been previously demonstrated that depletion of Mg^2+^ in the glutamate-containing medium may cause NMDARs activation, leading to cytotoxicity [28]. Similarly, chronic acid load induced upregulation of NBCn1 is associated with elevated glutamate-mediated toxicity, causing neuronal damage. Furthermore, acidosis leads to ATP depletion and membrane depolarization. Eliminating Mg^2+^, without Mg^2+^ blocking NMDARs, would induce more severe neuronal death [33]. Interestingly, NBCn1 knockout rodents show reduced glutamate-mediated neurotoxicity in hippocampal neuronal cultures. Under Mg^2+^-free conditions, they attenuate glutamate-induced neuronal death [40]. NMDARs stimulation was also studied in NBCn1 knockout mice and was found to be less sensitive to NMDA-induced seizures and neurotoxicity in the hippocampus. These studies provide in vivo and in vitro evidence that NBCn1 can serve as a novel neuroprotective target for glutamate-induced brain injury [42]. In addition to their role in neurotoxicity, NMDARs influence other cognitive and emotional processes, including learning and memory [43]. These receptor features lead researchers to speculate whether NBCn1 knockout animals had impaired learning and emotional states, notably anxiety.

Defects in vision and hearing may influence emotional and cognitive abilities [44,45]. NBCn1 is involved in maintaining hearing and vision function, which is elucidated by NBCn1 knockout mice. Three lines of NBCn1 knockout mice that investigate hearing and vision abnormalities have been reported. The 148 bp deletion in exon 5 causing auditory and visual defects generated the first relevant knockout mice. After one year of light stimulation, these mice lose 90% of the retinal activity caused by electrical impulses, resulting in photoreceptor degeneration [46]. In addition, these animals develop hearing disabilities due to morphological alterations of the cochlear duct and hair cell degeneration in the core component of the cochlea, the organ of Corti [47]. The second line of knockout mice was generated by integrating a gene trap vector into intronic sequences among exon 3 and exon 4, resulting in a reduction in NBCn1 protein expression by up to 70%. These animals have no phenotypic details but have normal viability [30]. The third line of knockout mice was generated by inactivating a promoter location in which a gene trap vector was inserted 434 bases upstream of the MEAD start codon. When compared to prior reports of selective photoreceptor loss and blindness induced by the first line of knockout mice, these animals had normal vision, and their hearing was impaired [48]. Since the third line of knockout mice was produced by a gene trap vector inserted in promotor P1, the difference in visual and auditory functions may be attributed to the differential expression of two NBCn1 variants in the retina and the cochlea. Astonishingly, these mice show no difference in spatial learning despite developing NMDAR downregulation. It will be interesting to test in future studies whether selective and complete deletion of NBCn1 in the hippocampus leads to learning and memory deficits.

As mentioned earlier, low pHi leads to a more severe neuronal death induced by the participation of NMDARs in cytotoxicity. However, it is unclear whether pH modulates the feeling of reward, the level of motivation, or the intensity of addiction. In the male NBCn1 knockout mice, the neurons have higher action potential thresholds and more depolarized membrane potentials, resulting in reduced membrane excitability, and exhibit lower pHi in comparison with wild-type controls [49]. In alcohol addiction experiments, NBCn1 loss contributes to elevated alcohol consumption and increased susceptibility to alcohol-induced sedation, which is attributed to intracellular acidosis and following reduced neural excitability. In addition, NBCn1 expression was reduced in the hippocampus and striatal neurons after chronic alcohol consumption. Therefore, chronic alcohol consumption promotes greater alcohol consumption in mice by reducing the expression of NBCn1 in a positive feedback mechanism, suggesting a vital role for NBCn1 in regulating alcohol consumption and susceptibility to alcohol-induced sedation [49,50].

As investigation concerning NBCn1 in the central nervous system has progressed, it has become clear that NBCn1 plays a vital role in physiologically regulating ion homeostasis and cellular signaling. NBCn1 is involved in the development and progression of brain injury and neurological diseases, and a better understanding of NBCn1 in neurological diseases will reveal its potential as a therapeutic target.

### 3.2. Role of NBCn1 in the Cardiovascular System

The NBCn1 has been recognized to be widely expressed in the cardiovascular system [51]. Using an antibody against the Nt sequence of an NBCn1 variant, researchers have repeatedly found that NBCn1 is expressed in endothelial cells and smooth muscle cells, whereas expression in cardiomyocytes remains controversial [27]. Recent studies have demonstrated the presence of NBCn1 in the ventricle by detecting a single band by Western blotting [52,53]. Immunolocalization investigations have also shown that NBCn1 is found in t-tubules, the lateral sarcolemma, and intercalated discs [52]. The activity of NBCn1 is regulated by multiple mechanisms, including phosphorylation and Ca^2+^-binding. The calcineurin binds to splice cassette II in the intracellular NH_2_-terminal domain of NBCn1 and activates NBCn1 transport activity upon elevation of intracellular Ca^2+^ [54]. In vasoconstriction, Ca^2+^-dependent stimulation of net acid extrusion through NBCn1 aids in the removal of the intracellular acid load associated with contraction and lowers intracellular acidification. Whereas the specific pathways involved in contractile-associated acid loading are not fully understood, it may entail an increase in metabolic acid secretion or cellular absorption of H^+^ transported by the cytoplasm Ca^2+^ ATPase. A significant function for NBCn1 may be seen in the resistance arteries of the cardiovascular system. Disruption of NBCn1 expression leads to low steady-state pHi in cells, which impacts local signaling in the arterial wall during vasodilation and contraction [32,55,56,57]. In endothelial cells, nitric oxide (NO) production from arteries was reduced in NBCn1 knockout mice, which was likely attributable to the direct effect of low pHi on endothelial NO synthase (eNOS), which has been shown to be strongly intrinsically pH sensitive in vitro [58]. This implies that NBCn1 expression in endothelial cells can have an effect on the production of eNOS. When mice were given the eNOS inhibitor N-nitro-L-arginine methyl ester (L-NAME), the production of lower NO resulted in decreased vasodilation and moderately increased blood pressure. Consistent with a role for reduced NO production in NBCn1 knockout mice.

In vascular smooth muscle, rho-kinase-dependent Ca^2+^ sensitivity is reduced in isolated resistant arteries of NBCn1 knockout mice, leading to lower contractility to norepinephrine stimulation after endothelial function inhibition [59,60,61]. Similarly, an altered arterial function is linked to about 2/3 suppression of blood pressure elevation after chronic Ang-II infusion in comparison with wild-type mic e [58]. However, no differences in pHi, vasodilator, or systolic function were observed in studies of wild-type and NBCn1 knockout mice in the absence of CO_2_/HCO_3_^−^, suggesting that the vascular effects of NBCn1 knockout are secondary to low pHi. The impact of the NBCn1 mutation on the development of arterial tone and blood pressure is complicated and depends on activity in the signaling pathway. At rest, NBCn1 knockout mice exhibit mild hypertension, consistent with the primary role of endothelial cell NO production described above, but NBCn1 knockout mice protected against Ang-II induced hypertension, suggesting that NBCn1 deletion attenuates the blood pressure response to NO synthase and rho kinase inhibition [62]. Reduced endothelial NO production and inhibition of the rho kinase signaling pathway are the underlying mechanisms of the altered arterial function that may lead to hypertrophy. Thus, inhibition of NBCn1 activity may represent a novel therapeutic strategy for the treatment of cardiovascular disease.

### 3.3. Role of NBCn1 in the Digestive System

NBCn1 is primarily found in the salivary glands, gastrointestinal tract, and pancreas. The NBCn1 in the saliva is essential for maintaining oral health, normal HCO_3_^−^ production neutralizes harmful acids produced by oral bacteria, thus preventing oral infections [63,64]. NBCn1 is abundant in the parotid and submandibular glands (SMG), which continuously transports HCO_3_^−^ to neutralize intracellular acidification, showing that it is an important molecular mechanism for fluid secretion processes in parotid cells. Numerous Studies have shown that NBCn1 has little role in stimulating HCO_3_^−^ secretion and pHi regulation in submandibular duct cells, and it can be speculated that much of the intracellular pH regulation occurs primarily through an unknown Na^+^ dependent transport mechanism, the specific mechanism of which is currently unknown.

NBCn1 is considered an important protective factor against gastric acid, expressed in the smooth muscle cell layer throughout the wall of the gastrointestinal tract, and is essential for protecting the mucosa from gastric acid delivery [28,30,32]. Furthermore, NBCn1 shows a distinct distribution in the mice’s intestinal tract. The level of NBCn1 expression is much lower in the jejunal and ileal mucosae compared to the duodenal and colonic enterocytes [30]. The pH of gastric contents emptied into the duodenum can occasionally reach as low as 1. The potential mechanism for duodenal protection against the acid load is through secreting HCO_3_^−^ into the lumen, thus neutralizing H^+^ that diffuses through the mucus gel. The expression of NBCn1 on the basolateral membrane [28] of the duodenal mucosa indicates its significant role in transepithelial HCO_3_^−^ secretion, which is critical for protecting the mucosa from stomach acid. It has been shown that a dysfunctional mucus barrier causes a risk for pathogen-mediated mucosal injury in the colon, which may be triggered by both normal and pathogenic forms of intestinal flora [65]. In a study reporting whether NBCn1 affects HCO_3_^−^ secretion rates, NBCn1 knockout mice were found to be unable to maintain normal HCO_3_^−^ secretion rates, which indicates that NBCn1 acts as a major pHi regulatory mechanism in mouse duodenal cells [66,67]. Therefore, more information on NBCn1 in the gastrointestinal tract is needed to design pharmacological strategies.

### 3.4. Role of NBCn1 in the Kidneys

There has been substantial uncertainty over the subcellular localization of NBCn1; however, its expression is very prevalent in collecting duct intercalated cells in the inner stripe of the outer medulla and in the inner medulla [68]. This ambiguity in cellular expression of NBCn1 may be due to various splice variants, and it has impeded the development of a hypothesis about its physiological role in the reabsorption or secretion of acid–base equivalents in the collecting duct. Under acidic conditions, HCO_3_^−^ extrusion is restricted, and NH_4_^+^ entering the medullary thick ascending limbs (mTAL) cells through furosemide-sensitive NKCC2 transporters or ROMK channel might dissociate into NH_4_^+^ and NH_3_ [68]. The generated NH_3_ may preferentially exit the cell by nonionic diffusion through the basolateral membrane and finally be transported into the acidic compartment of the collecting duct. The remaining proton may be transported directly through the Na^+^-H^+^ antiporter and/or buffered by imported HCO_3_^−^. The subsequent production of carbon dioxide would allow recovery of HCO_3_^−^ at the basolateral membrane while stimulating NH_4_^+^/NH_3_ uptake by buffering intracellular H^+^ load. However, the H^+^ overload caused by increased NH_4_^+,^ in turn, stimulates NBCn1 upregulation [37,69]. This demonstrates that NBCn1-mediated HCO_3_^−^ uptake is an important event in NH_4_^+^ cellular transport. It is certain that NH_4_^+^ is the main H^+^ carrier for renal acid excretion, and the function of NBCn1 may be associated with the recycling of NH_4_^+^.

In vivo animal studies showed a nearly 10-fold increase in NBCn1 protein expression in thick collecting duct [69]. NBCn1 is down-regulated in metabolic alkalosis and upregulated in metabolic acidosis due to increased H^+^ overload caused by increased NH_4_^+^ uptake [70,71]. Furthermore, NBCn1 is thought to generate and maintain the NH_4_^+^ gradient required for urinary concentration and excretion, indicating the importance of NBCn1 in urinary acidification [72].

## 4. Physiological Role of NBCn1 in Breast Cancer

**Figure 4:**
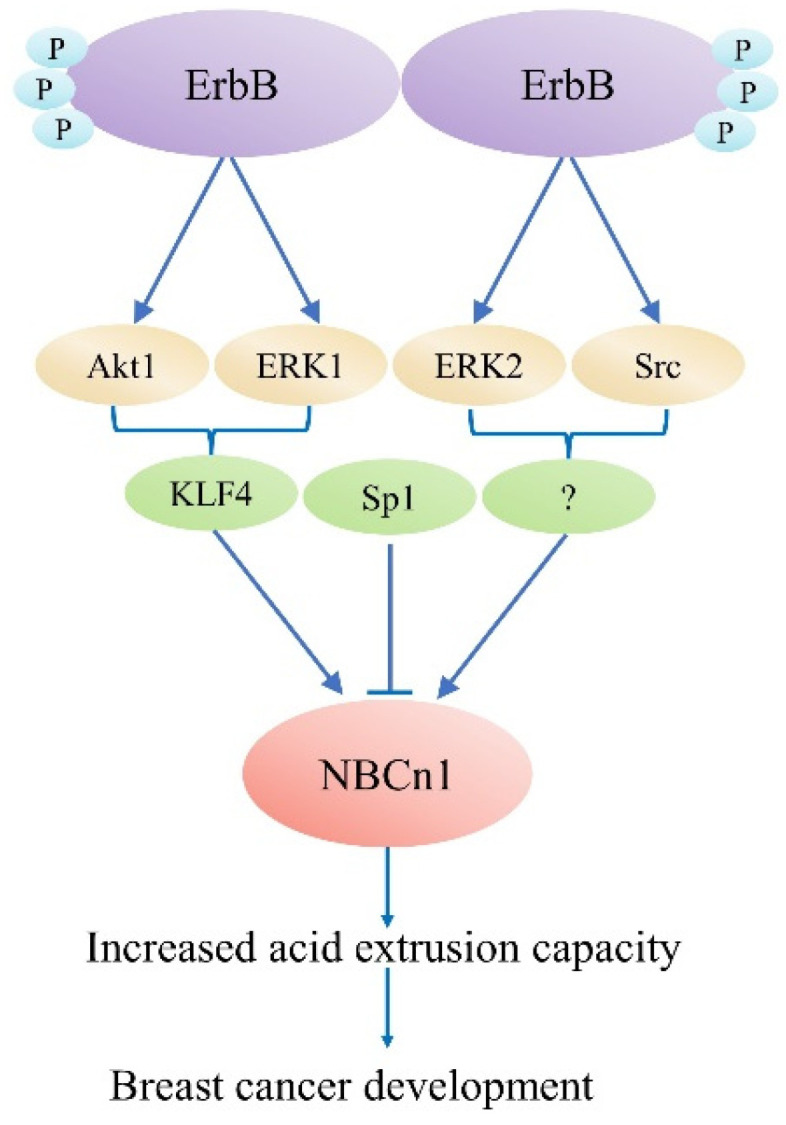
ErbB2-dependent SLC4A7 expression regulation model. The truncated ErbB2 receptor is responsible for regulating SLC4A7 expression.

Experimental evidence supports the function of acid–base transports in cancer [73], while the function of NBCn1 in cancer and the pharmacological potential of inhibiting this transporter is still unclear. Recently, it was discovered that the presence of Nt truncated ErbB2 receptor (NErbB2) in the cancer cell line MCF-7 significantly upregulated NBCn1 expression. This overexpression increases cancer cells’ acid excretion ability, allowing them to lower the enormous intracellular acid burden induced by glycolytic metabolism and regulate pHi. This might work by the following mechanism: (1) full-length ErbB2 stimulates NBCn1 via the MEK/ERK1/2 and/or PI3K/AKT pathways; (2) The NErbB2 receptor activates Kruppel-like factor 4 (KLF4) through MEK/ERK1and/or PI3K/AKT1 pathways bind the NBCn1 promoter and increase mRNA and protein expression; and (3) NErbB2 promotes MEK/ERK2 and/or (Src) kinase to increase NBCn1 expression. Downregulation of another KLF4 superfamily member, Sp1, reduced NBCn1 via the aforementioned pathways, although the mechanism is unclear [74], as shown in Figure 4. This study indicated a substantial role for NBCn1 in ErbB2/HER2-positive breast cancers. With siRNA-knockdown investigations and pharmacologic inhibition, researchers found that NBCn1 is liable for the extrusion of acid in MCF-7 cells [73].

Several recent genome-wide association studies have shown a correlation between breast cancer and genomic polymorphism in NBCn1, which raises the intriguing possibility that NBCn1 contributes to the etiology of human cancer [75,76,77]. Homozygosity for the single nucleotide polymorphism (SNP) rs4973768 was shown to increase the risk of breast cancer by 1.2–1.3 times [75,77]. A deeper study at the site of SNP suggests that it may modify the binding affinity for the microRNA miR-569, whose function in modulating NBCn1 expression levels is under investigation. NBCn1 contributes to breast cancer or acts as a modulator of cancer prognosis, as revealed by the finding that rs4973768 SNP is more common in breast cancer patients than in healthy controls. Recently, research identified that NBCn1 acts as a tyrosine kinase substrate during the progression of cancer [78], providing more evidence for the notion of NBCn1 in breast cancer. Remarkably, evidence from the MCF10AT family of breast cancer cell lines revealed that NBCn1 expression would decrease rather than increase as the disease progressed. Additional research, especially in tumor samples and in vivo, is required to define the significance of acid–base transporters.

## 5. Pharmacological Roles of NBCn1

**Figure 5:**
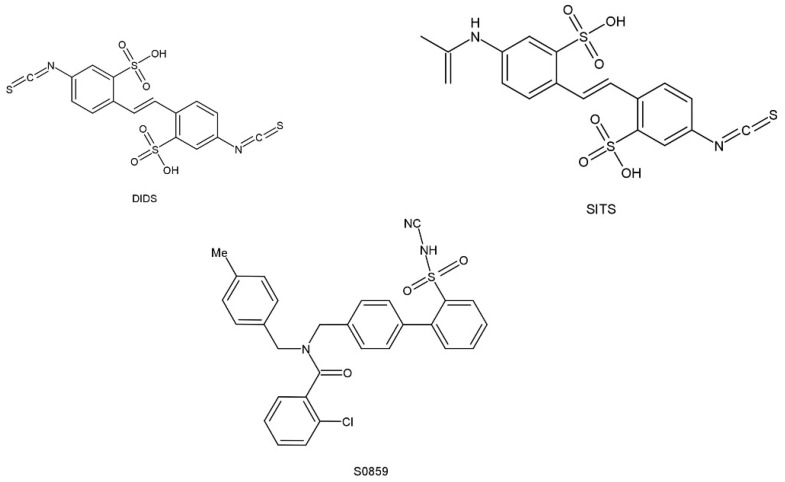
Chemical structural formula of NBCs inhibitor.

Several different research organizations have been on the seek for pharmacological substances in order to conduct experiments that will identify the regulatory functions of NBCn1 cotransporters and for possible use in the treatment of disease. Some inhibitors of NBCn1 cotransport activity have been obtained, but these may be NBCs inhibitors rather than specific inhibitors of NBCn1. The lack of specificity prevents them from being widely used. In addition, these inhibitors may be used in experiments to monitor cellular function or to inhibit additional ion transport proteins (e.g., DIDS), which can make experiments difficult. However, these NBCs inhibitors can provide useful information on the molecular structure, which can interact with the NBCs cotransport proteins and may act as the initial point for the development of more specific therapeutic tools. The structure of these inhibitors is shown in Figure 5. Stilbene derivatives (e.g., DIDS, SITS) are extensively used NBCs inhibitors. However, the role of DIDS is more complex in the case of NBCn1. It was found that NBCn1 was unresponsive to DIDS even in the high micromolar range in mice mesenteric artery endothelial cells, as well as in choroid plexus and renal cells, while it was completely inhibited by DIDS in rat arterial smooth muscle cells. It might be due to the lack of an intact DIDS motif in NBCn1, resulting in lower DIDS sensitivity in certain cell types. To test this hypothesis, related RT-PCR products from NBCn1 expressed in mice mesenteric arteries were sequenced and reported to lack a complete DIDS motif [59]. Furthermore, the DIDS sensitivity of NBCn1 is enhanced when the KLFH sequence is mutated to KLFK [79].

S0859, an N-cyanosulfonamide compound that acts as a generic NBCs inhibitor, is unable to distinguish between different Na^+^/HCO_3_^−^ cotransport isoforms but shows significant improvement over stilbene derivatives. The preferred dose of S0859 in vitro was 15 μM, which provided about 90% inhibition of NBCs activity. Recently, S0859 exhibited inhibition of NBCn1 activity in MCF-7 human breast cancer cell lines [73]. S0859 is selective in acid–base transport in ventricular cardiomyocytes, where it does not impact Cl^−^/HCO_3_^−^, Cl^−^/OH^−^, or Na^+^/H^+^ exchange [80].

In addition, the application of inhibitory antibodies may be an effective way to inhibit NBCn1. Two investigations suggest antibodies may regulate Na^+^/HCO_3_^−^ cotransport activity. In one research, NBCn1 activity in rat ventricular cardiomyocytes was successfully inhibited by a rabbit polyclonal antibody [81]. In another investigation, two antibodies against NBCe1 were prepared. One of these antibodies was able to suppress NBCe1 activity in cat ventricular cardiomyocytes, whereas the other antibody appeared to promote NBCe1 activity [82]. Antibodies that target specific proteins on cancer cells (such as anti-HER2 receptor antibodies) are effective in human clinical trials, providing a reason to believe that acid–base transporter inhibitory antibodies may find their way into clinical usage.

## 6. Conclusions

Despite the potential significance of NBCs, the present understanding of multiple molecular activities, distribution, regulation, physiological functions, and pathological roles is very limited. In this review, we explored the basic molecular, pharmacological, and physiological properties of NBCn1 as well as advanced investigations into their significance in cancer. Results from NBCn1 knockout mice have shed light on the role in tissue physiology and pathologies such as epilepsy and ischemia/hypoxia. The significance of NBCs transporters to particular cancer types is unclear; however, evidence suggests NBCn1 may be crucial for cancer cells. Although a disrupted NBCn1 is linked to conditions such as cancer, heart disease, and metabolic problems, the particular physiological function, as well as the prospective clinical applications, are not yet fully investigated. As the molecular mechanisms of NBCn1 are gradually clarified, research and development of targeted drugs should be conducted for NBCn1 and its related pathways.

## Figures and Tables

**Table 1:** SLC4-linked HCO_3_^−^ transporter-related diseases.

Gene	Mutant Types	Function	Disease
SLC4A4(NBCe1) [11]	Nonsense mutation; missense mutation; frame-shift mutation; 3′UTR mutation; heterozygous mutation	Intracellular retention and truncation of all NBCe variants	Proximal renal tubular acidosis with ocular abnormalities (pRTA) Mental retardation Dental and ocular abnormalities Hemiplegic migraine Epilepsy
SLC4A5(NBCe2) [12,13]	Splicing mutation	Involved in the regulation of acid–base balance and the regulation of blood pressure	Arterial hypertension Renal metabolic acidosis Inherited retinal disease
SLC4A7(NBCn1) [14,15]	Homozygous frame-shift mutation	Modulates pHi and bicarbonate levels	Night blindness Hypertension Epilepsy Breast cancer
SLC4A10(NBCn2) [16]	Nonsense mutation	Control of neuronal excitability	Epilepsy Mental retardation
SLC4A8(NDCBE) [8]	ND	NA	Epilepsy

Abbreviation: ND; not determined, NA; not available.

## Data Availability

Not applicable.
